# Attraction of *Rhodnius prolixus* males to a synthetic female-pheromone blend

**DOI:** 10.1186/s13071-018-2997-z

**Published:** 2018-07-16

**Authors:** Björn Bohman, Alyssa M. Weinstein, C. Rikard Unelius, Marcelo G. Lorenzo

**Affiliations:** 1 School of Molecular Sciences, the University of Western Australia, Perth, Australia; 2 Reseach School of Biology, the Australian National University, Canberra, Australia; 3 Faculty of Health and Life Sciences, Linneaus University, Kalmar, Sweden; 4 Grupo de Comportamento de Vetores e Interação com Patógenos, Instituto René Rachou/FIOCRUZ, 30.190-002, Belo Horizonte, Minas Gerais Brazil

**Keywords:** *Rhodnius prolixus*, Chagas disease, SSR, Olfactometer, Pheromone, Metasternal gland, Trap, Volatiles

## Abstract

**Background:**

The triatomine bug *Rhodnius prolixus* Stål, 1859 (Heteroptera: Reduviidae) is the primary vector of Chagas disease in Colombia and Venezuela. An important step in controlling Chagas disease is monitoring the growth and spread of bug populations to inform effective management. Such monitoring could be carried out using pheromone traps. To develop effective pheromone traps, it is vital to understand the pheromone chemistry of *R. prolixus*. Previous studies have found that female *R. prolixus* metasternal gland secretions induce males to: leave shelters, take off, orientate during walking, aggregate around mating pairs, and mate. This study aims to identify a synthetic blend of female metasternal gland compounds that could be used to attract *R. prolixus.*

**Results:**

We investigated the electrophysiological activity of the ten most abundant compounds in female *R. prolixus* metasternal glands using single sensillum recordings. In total we obtained 60 recordings from basiconic sensilla in male *R. prolixus*. In 31 of these recordings, responses to individual compounds were observed. Each of the ten tested compounds elicited neuron responses in a minimum of eight recordings. Having confirmed their electrophysiological activity, we tested these ten compounds by presenting them to male *Rhodnius prolixus* in a “T” olfactometer. Male bugs showed a significant preference for the blend of metasternal gland compounds compared to the clean air control.

**Conclusions:**

A simple blend of ten compounds found in female *R. prolixus* metasternal glands is attractive to conspecific males. All compounds in the blend are either commercially available at low cost, or easily synthetically prepared from simple precursors. We hope that this blend will be evaluated as a lure for pheromone traps in field bioassays.

**Electronic supplementary material:**

The online version of this article (10.1186/s13071-018-2997-z) contains supplementary material, which is available to authorized users.

## Background

The triatomine bug *Rhodnius prolixus* Stål, 1859 (Heteroptera: Reduviidae) is the main vector of Chagas disease in Colombia and Venezuela [1, 2]. Triatomine bugs mainly occur in temperate and tropical areas of the Americas, ranging from southern USA to central Argentina and Chile. While housing improvement programs and investment in pest control have largely eradicated triatomine populations in several regions [2], the bugs continue to pose a problem in many underprivileged communities.

Studying triatomine pheromone chemistry is crucial to the development of pheromone bait traps, which are used to monitor the growth and spread of bug populations. Monitoring can inform effective investment in population control, and thereby limit the spread of Chagas disease. Short-range monitoring pheromone bait traps could be installed in dwellings at risk, for example those from which bugs had recently been killed by insecticide treatment. With adequate development, pheromone bait traps may also present an alternative to current control measures [3–5]. Triatomine bug populations are commonly controlled by insecticide application in infested houses and their peridomiciles. The efficacy of this control measure is hampered because several populations of the two main Chagas disease vectors, *Triatoma infestans* and *R. prolixus*, have developed insecticide resistance [6–8]. Using a sexual pheromone bait, mass trapping and or attract-and-kill traps could be developed to reduce populations of triatomine males, which in turn, would reduce the number of offspring produced.

The study of triatomine pheromones began over 40 years ago, with the discovery that copulating pairs of *R. prolixus* emit a pheromone that is attractive to males [9]. It was subsequently proposed that the source of triatomine sexual pheromones may be the metasternal glands, which were suggested to be involved in sexual communication in *Triatoma infestans* [10]. Several highly volatile ketones and alcohols produced in *T. infestans* metasternal glands were identified, with 3-pentanone being shown to be emitted by adult *T. infestans* during copulation [10]. Similar ketones and alcohols were also recently found in the metasternal glands of *T. longipennis*, *T. palidipennis* and *T. phyllosoma* [11]. Metasternal gland secretions were first shown to be behaviourally active in *T. brasiliensis*, as males oriented towards female metasternal gland odour in olfactometry experiments [12]. Metasternal gland secretions have subsequently been found to induce *Rhodnius prolixus* males to: leave shelters [13], take off [14], orientate during walking [15], aggregate around mating pairs [16], and mate [15, 16]. The 12 most abundant compounds present in female *R. prolixus* metasternal gland secretions have been identified [15]; however, the biological activity of these isolated compounds has remained unknown until now.

Electrophysiological methods such as electroantennogram detection (EAD or EAG) and single sensillum recording (SSR) can be implemented to investigate which chemical volatiles may function as pheromones in an organism. In triatomine bugs, such methods have been used to study pheromones in surprisingly few cases (see [5] for a review). Three morphological types of chemosensilla have been described in triatomines: trichoid, basiconic and grooved-peg sensilla [5]. There is a substantial increase in the number of basiconic (and to a lesser extent trichoid) sensilla following the imaginal moult in *R. prolixus* [17, 18]. Basiconic sensilla have more than 15 neurons at their base, indicating their potential to detect a diverse range of odours [19]. The greater number of basiconic sensilla possessed by *R. prolixus* adults compared to larvae, in combination with the large number of neurons housed within them, may indicate that these sensilla are involved in sexual communication. In fact, adults show a substantial increase in the expression of odorant and ionotropic receptor genes in their antennae compared to 5th instar larvae [20]. However, to date, most reports on triatomine responses to odours have focused on grooved-peg rather than basiconic sensilla [21, 22]. Additionally, no reports of SSR experiments using *R. prolixus* were found in the literature.

In the present study, we worked towards the development of a sexual pheromone bait that could be used in controlling the Chagas vector *R. prolixus.*

Specifically, we aimed to investigate the electrophysiological responses to the ten most abundant compounds in the metasternal glands of *R. prolixus* females (as identified in [15]) by SSR. Subsequently, we aimed to determine whether this ten-compound blend was attractive to *R. prolixus* males in olfactometer experiments.

## Methods

### Insects

*Rhodnius prolixus* were reared under laboratory conditions at 26 ± 2 °C and 60 ± 10% relative humidity. Bugs were fed on citrated rabbit blood using an artificial feeder strictly following the FIOCRUZ ethical regulations (CEUA-FIOCRUZ-MG license number LW-61/2012). Fifth-instar larvae were sorted by sex (following [23]) into plastic containers containing a shelter made of fluted filter papers. Adult bugs were fed when 15 days-old, and tested in experiments when 25 ± 5 days-old. All bugs were kept at a 12:12 light/dark cycle for a minimum of three days before they were used in experiments. All experiments were conducted during the first five hours of the scotophase. These conditions were selected as adult triatomine bugs kept in similar conditions have been found to readily mate [12, 24].

### Chemicals

**Fig. 1:**
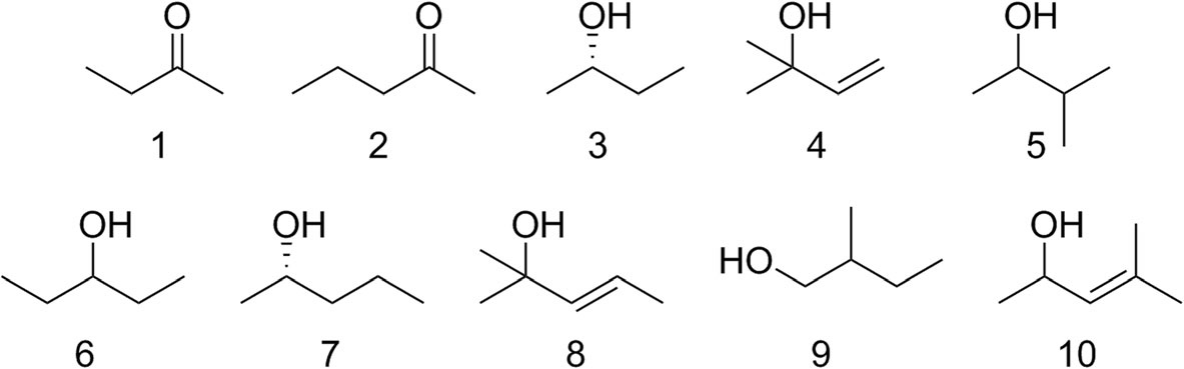
Structures of all compounds in Table 1 from the metasternal glands of female *Rhodnius prolixus*

All compounds were obtained or synthesized as described previously [15]. A synthetic blend of the ten most abundant of these compounds was prepared based on the relative amounts previously identified from female glands (see Fig. 1 and Table 1).

**Table 1:** Compounds (with concentration in mM) in synthetic blend, based on previous analysis of the metasternal glands of female *Rhodnius prolixus* [12]

Compound number	Compound name	Concentration in blend (mM)
1	2-Butanone	0.5
2	2-Pentanone	0.5
3	(*S*)-2-Butanol	5.7
4	2-Methyl-3-buten-2-ol	61
5	3-Methyl-2-butanol	1.1
6	3-Pentanol	5.4
7	(*S*)-2-Pentanol	20
8	(*E*)-2-Methyl-3-penten-2-ol	6.1
9	2-Methyl-1-butanol	3.5
10	4-Methyl-3-penten-2-ol	2.1
	Total blend	100

### Single sensillum recordings

Adult male *R. prolixus* were mounted with the ventral side down on a microscope slide, with the antennae positioned parallel to the horizontal edges of the slide. To immobilize bugs during mounting, they were placed in a freezer for 3–4 min immediately prior to mounting. Bugs’ legs and rostrum were fixed onto the slide with double-sided transparent tape and a small piece of periphery wax was used to hold their heads. A glass coverslip covered with double-sided tape was inserted under the antennae, and fixed to the top of the slide with periphery wax. The antennae were gently fixed to the doubled-sided tape beneath them using a soft brush made of a single eyelash fixed to the tip of a glass capillary tube. To reduce antennal movement during recordings, the extremes of the distal segments of the antennae were fixed to the coverslip with an additional small piece of tape.

A reference electrode, consisting of a sharpened stainless-steel needle mounted into a Syntech electrode holder, was inserted at right angles into the left eye of the bug. The needle tip was pressed against the eye with a manual micromanipulator and the head was gently pushed towards the needle tip with a blunt palm needle until a firm contact was established. Using an Olympus BX51W1 microscope (Tokyo, Japan), a sharp recording tungsten electrode was inserted into the base of a basiconic sensillum with a Märzhäuser DC-3KS micromanipulator. The electrode approach was at a 30–45° angle to the antennal surface. SSR experiments were conducted on basiconic sensilla in accordance with our hypothesis that they may be involved in the detection of sexual signals due to their increased number in the adult stage [17, 18].

The charcoal-filtered, humidified (DI water) airflow was controlled by a Syntech stimulus controller CS-55 (Kirchzarten, Germany), with setting “5” for both continuous flow and pulse flow. The flow was measured at 0.25 m/s at the end of the glass tube, which had a 10 mm inner diameter.

The ten most abundant compounds previously identified [15], were tested for activity on each sensilla both as a blend and individually. A host-related odour, octanal, was also tested on each sensilla, as this compound has been reported as active in some basiconic sensilla of *T. infestans* [22]. The solvent dichloromethane was used as a negative control. All compounds were prepared in dichloromethane as 0.1 M solutions. Each compound/blend/control (10 μl = 1 μmol) was dispensed onto a filter paper (Whatman Grade 1; 1 cm^2^) placed in the upper end of a Pasteur pipette (150 mm) which was used as the odour source. The tip of each Pasteur pipette was inserted into the airflow tube through a small hole. For each sensillum, the ten-compound blend (Table 1) was tested first, and if a spike burst was detected, the compounds, dichloromethane control, and octanal were then tested individually at 3 min intervals [25] in a randomized order. The SSR-signals were recorded for 6.0 s, starting 2.0 s before stimulation.

A Syntech Universal AC/DC probe was used to record the signal (amplified 10×) captured by the tungsten electrode. This signal was fed to a PC *via* a Syntech IDAC 4 amplifier. Autospike software (Syntech, v.3.9) was used to record and analyse all data. The sampling rate was set to 2400 Hz, the pre-trigger to 2 s, and the pulse duration to 1 s. A filtering range of 200 Hz to 2 kHz was applied, and 50/60 Hz suppression was enabled. The response to stimulus-presentation was measured as the number of spikes from 0.5–1.5 s after the onset of stimulation. The background activity was measured as the number of spikes 1.0–0 s before the onset of stimulation. The net number of spikes was defined as the subtraction of background activity from the response to stimulation. It is noteworthy that in most cases neurons were virtually silent prior to the stimulus. We therefore chose to describe a neuron as excitable by a tested odour if the number of spikes increased by > 10 counts/s after stimulation, and was > 25% of the number of spikes elicited by the most active compound in that series.

### Olfactometer

A simultaneous two-choice “T” olfactometer was used to test the orientation responses of adult males to the synthetic blend of female metasternal gland compounds. The olfactometer was made of poly(methyl methacrylate) (two 21.5 cm arms and a 33.5 cm common stem, both rectangular, arm section 24.0 cm^2^) [12]. The apparatus was positioned horizontally, on foam cushions to minimize vibrations, in an experimental room under the same temperature conditions as described for the insect rearing. A piece of filter paper covered the floor of the olfactometer to allow the insects to walk easily. Filtered air was pumped through the olfactometer using an aquarium pump, with the airflow being split through a glass “T” piece into two separate streams: one for each arm. The air-flow was maintained at a speed of 0.01 m/s at the release chamber and 0.04 m/s at the base of each arm, as measured by an anemometer (Testo, Lenzkirch, Germany). Each arm was connected to a small polyacrylic chamber (6.0 × 6.0 × 3.5 cm) in which female bugs were presented as odour sources. For the blend-testing assays, an aliquot of the blend (or dichloromethane control stimulus) was applied onto a filter paper (1 cm^2^), which was inserted into a Pasteur pipette that was connected with silicon tubing to the olfactometer stimulus box. The stimuli were alternated between the olfactometer arms in each assay.

Assays were conducted in a darkened experimental room. To allow the behaviour of the bugs to be observed without disturbance, an infrared sensitive CCD video camera was set up. To ensure bugs were not agitated during experiments, bugs were gently separated from their colonies in dimmed light. Furthermore, after separation, bugs were left to acclimate in individual Petri dishes for a minimum of 20 min before introduction into the olfactometer. After being placed into the release chamber of the olfactometer, bugs were left to acclimate for a further 10 min. Subsequently, the air pump was turned on and the door separating the male from the choice chamber was gently opened. The behaviour of the insect was recorded until either (i) the bug made a choice, or (ii) a period of 15 min had expired. A choice was defined as the whole body of the bug passing from the choice chamber of the olfactometer into one of the two arms. The responses were analysed using a binomial test in R version 3.4.0 [26]. All bugs, both male and female, were used only once. To avoid any bias due to residual chemical contamination from walking males, the filter paper on the floor of the olfactometer was replaced, and the olfactometer wiped with water and left to dry, after each assay. Additionally, the olfactometer was cleaned with ethanol twice daily and dried for at least 30 min before new assays were conducted.

Three experiments were conducted in the olfactometer:

- Experiment A: to provide a negative control, we evaluated whether bugs exhibited an intrinsic preference for either arm of the olfactometer when presented with equal stimuli. *Rhodnius prolixus* adult males (*n* = 40) were tested with two clean air currents (no odour stimulus);
- Experiment B: to provide a positive control demonstrating that male bugs orient towards female odours in the olfactometer set-up, we evaluated the responses of *R. prolixus* males (*n* = 40) to sexually mature female bugs. Female bugs were placed in one stimulus box, while the alternate box was left empty. The arm in which the females were placed was alternated between assays. To control for individual variation in attractiveness, two female bugs were used as stimulus in each experiment. After each assay, the stimulus chamber was checked for faeces. Given that freshly deposited faeces have been reported to repel bugs [27, 28], results from assays in which females had defecated in the stimulus chamber were excluded;
- Experiment C: to investigate whether the previously identified blend of metasternal gland compounds from female *R. prolixus* induced male orientation, we evaluated the responses of *R. prolixus* males (*n* = 50) to a blend of synthetic metasternal gland compounds (Table 1). A dose of 10 μl of a 0.1 M solution (10 μl = combined amount of all compounds = 1.0 μmol) was used as the stimulus and dichloromethane (10 μl) was used as a control in the alternate arm.

## Results

### Single sensillum recordings

We investigated the electrophysiological activity of the ten most abundant compounds previously identified [15] to find candidate compounds to trial in the olfactometer experiment. Responses to the full blend of metasternal gland compounds (blend as in Table 1) were observed in 60 SSR recordings. In 31 of these recordings, confirmatory responses to single compounds, when tested individually, were observed. Throughout the study, each of the ten tested compounds elicited neuron responses in a minimum of eight recordings. The two isomers of pentanol (compounds **6** and **7**) most frequently elicited neuron responses, with a total of 15 and 16 recordings showing responses respectively.

Despite attempts to group the neurons based on spike amplitude and response to different classes of compounds, no meaningful groups could be formed as several interesting complexities were present:

(i) In most of the recordings (> 90%) the amplitude of the spikes from firing neurons was much larger after stimulation than during their previous spontaneous activity.
(ii) Many different spike amplitudes were detected after stimulation, indicating that many neurons were active in each sensillum.
(iii) The solvent control elicited an excitatory response in 16% of the recordings analysed.
(iv) In some recordings, in which spontaneous neuronal activity was observed, some or all of the compounds inhibited firing.

Examples of representative recordings showing excitatory and inhibitory responses are provided in Additional file 1.

Given these complexities, which are interesting for future work, and the limited number of standardized bugs available, we concluded that SSR currently would not expedite the optimization of the synthetic chemical blend. Therefore, the full blend of metasternal gland compounds was used in the olfactometer experiment.

### Olfactometer bioassays

**Fig. 2:**
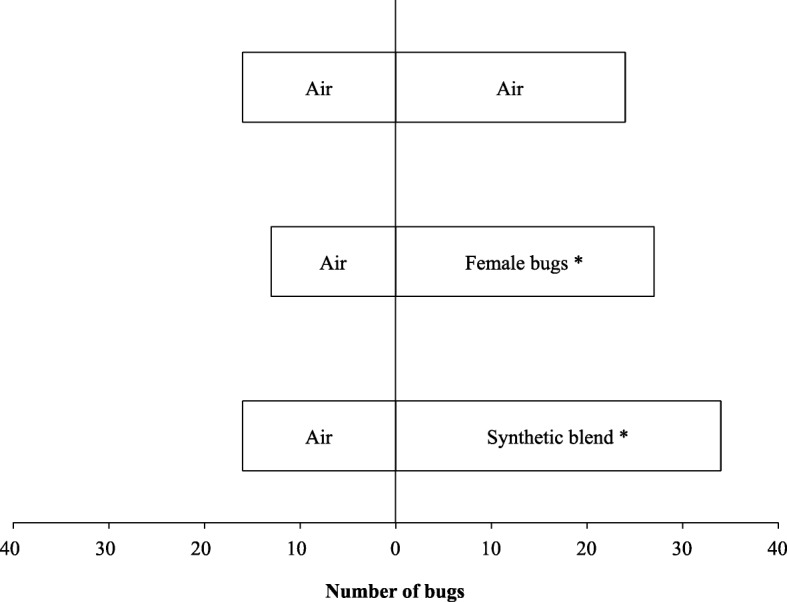
Bug choices in the two-way olfactometer in which bugs were presented with: two arms providing clean air; one arm providing female-emitted odour and the other clean air; and one arm providing synthetic odour blend and the other clean air. *P-value < 0.05 in a binomial test

In the negative control experiment A, male bugs did not show significant preference for either arm of the olfactometer when two clean air currents were presented [Fig. 2, *P* = 0.268, *n* = 40 (24 *vs* 16)]. In the positive control experiment B, the male bugs showed a significant preference for the live female odour [Fig. 2, *P* = 0.038, *n* = 40 (27 *vs* 13)]. Experiments A and B confirmed that the olfactometer did not show any bias between the two arms and that female odour did elicit attraction from male *R. prolixus,* confirming the adequacy of the setup for evaluating male responses to female odour*.* In experiment C, the male bugs showed a significant preference for the blend of synthetic metasternal gland compounds in comparison to the dichloromethane control [Fig. 2, *P* = 0.015, *n* = 50 (34 *vs* 16)].

## Discussion

The results from this study indicate that *R. prolixus* basiconic sensilla have olfactory receptor neurons (ORNs) that respond to female metasternal gland compounds. Given that the blend of female metasternal gland compounds elicited male attraction, and that the number of basiconic sensilla is known to increase with the onset of the adult life stage [17, 18], we suggest that these sensilla play a role in mediating sensory processes involved in sexual communication in this species. Our study represents one of the first forays into SSR in *R. prolixus*, and the complexities we encountered indicate that further research into this topic is both possible and warranted.

In our experiments on basiconic sensilla, we observed a range of neuron response profiles. Some sensilla presented inhibited neuron activity upon stimulation, some responded with increased spike frequency to a few specific compounds, while others responded to many compounds, including the host volatile octanal and in some cases even to dichloromethane. As a result, we were unable to group sensillar response profiles from the highly complex pattern of responses observed. This complexity may have been due to the large number of neurons suggested to be present in *R. prolixus* basiconic sensilla [19], making counting the spikes of individual ORN responses unfeasible.

We limit our conclusions about the electrophysiological activity of the individual compounds to stating that ORN responses were triggered by the puffing of all metasternal gland compounds tested in this study. As the two pentanol isomers (compounds **6** and **7**) were the most frequently active, these compounds should be considered as candidates for future work, together with 2-methyl-1-butanol (**9**), which, in addition to eliciting responses in almost half of our SSR recordings, was most frequently detected over copulating pairs in a previous study [15]. To be able to sort functional types of basiconic sensilla present in *R. prolixus*, highly replicated studies, including dose-response experiments, would be needed, and thus many more standardised bugs and recordings would additionally be required. In light of the complexities encountered in the SSR analyses and the limited number of standardised bugs to which we had access, priority was given to testing the full blend of compounds in the olfactometer bioassay.

The olfactometer assay confirmed male anemotaxic orientation mediated by sexual stimuli in *R. prolixus*, and supports the proposal that female triatomine bugs produce a sexual pheromone that attracts males from a distance [12]. More prominently, the olfactometer assay additionally demonstrated that a blend of *R. prolixus* female metasternal gland compounds can be used to attract males, and therefore that this blend has potential for use as a bait in a pheromone trap.

Given that the compounds used in the olfactometer experiment are all either commercially available or inexpensive to prepare, we suggest that at present this blend of compounds should be evaluated as a bait for traps in field bioassays, without the delay of preparing additional standardised bugs for subtractive olfactometer trials, or unravelling the full complexities of sensillar physiology. It should be noted that a multi-blend bait would require careful formulation when applied in field traps, as the physical properties, such as the volatility, would change the dynamics over time as the blend may change. In confirming the behavioural activity of the previously identified compounds [15], this study provides the crucial step after compound identification to justify the testing of these compounds in extensive field bioassays in remote areas of Colombia and Venezuela that are affected by *R prolixus-*transmitted Chagas disease.

## Conclusions

A blend of ten structurally simple compounds from the metasternal glands of female *R. prolixus* is attractive to *R. prolixus* males. Since all compounds in the blend are easily obtainable, we propose that this blend of compounds be evaluated as a lure in pheromone traps in the field.

## Additional file

Additional file 1: Selected SSR-traces. Two sets of SSR-recordings are presented to exemplify the observations reported in the Results section. (DOCX 1593 kb)

## Abbreviations

CCD: charge-coupled device
DI: deionized
EAD: electroantennographic detection
EAG: electroantennography
ORN: olfactory receptor neuron
SSR: single sensillum recording
