# Supplementary material for: Attraction of Rhodnius prolixus males to a synthetic female-pheromone blend
Source: Parasit Vectors. 2018 Jul 16;11:418. doi: 10.1186/s13071-018-2997-z (PMC6048742; doi:10.1186/s13071-018-2997-z)
Supplement: Supplementary file 1 — Selected SSR-traces. Two sets of SSR-recordings are presented to exemplify the observations reported in the Results section. (DOCX 1593 kb) [file 13071_2018_2997_MOESM1_ESM.docx]

Attraction of *Rhodnius prolixus* males to a synthetic female pheromone blend

Björn Bohman, Alyssa M. Weinstein, C. Rikard Unelius, and
Marcelo G. Lorenzo

**Supplementary Information**

**Selected SSR-traces**

Two sets of SSR-recordings are presented to exemplify the observations reported in the results section:

1. In most of the recordings (>90%) the amplitude of the spikes from firing neurons was much larger after stimulation than in the spontaneous activity of unstimulated neurons.

*Examples: Trace 1.*

2. Many different spike amplitudes were detected after stimulation, indicating that many neurons were active in each sensillum.

*Examples: Traces 1 and 2 (also see magnifications on page 2).*

3. The solvent control elicited a response in 16% of the recordings analyzed.

*Examples: Trace 1 and 2.*

4. In some recordings, in which spontaneous neuronal activity was observed, some or all of the compounds inhibited firing

*Example: Trace 2 (also see magnification on page 2).*

**Trace 1**

**
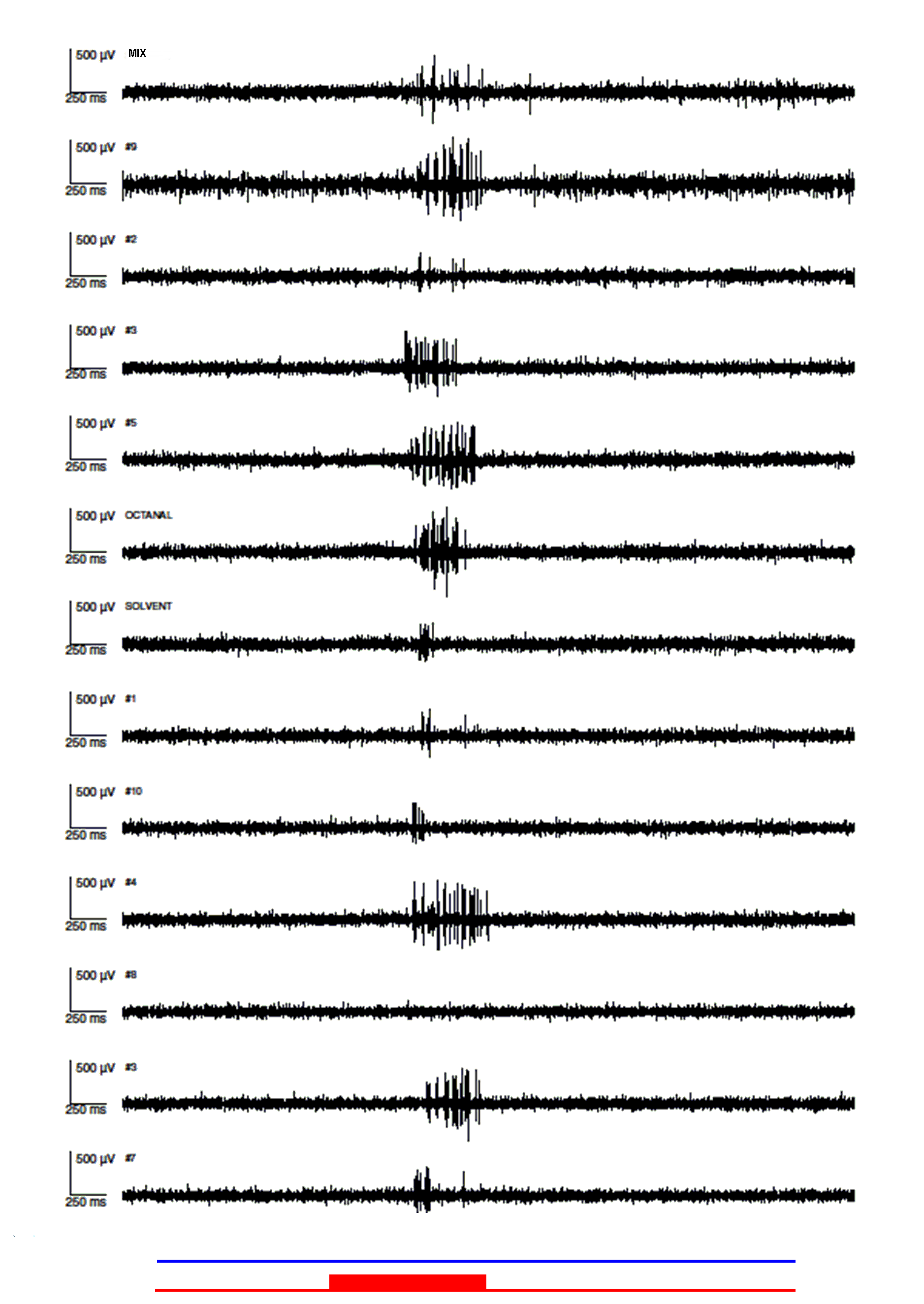
**

**Trace 2**

**
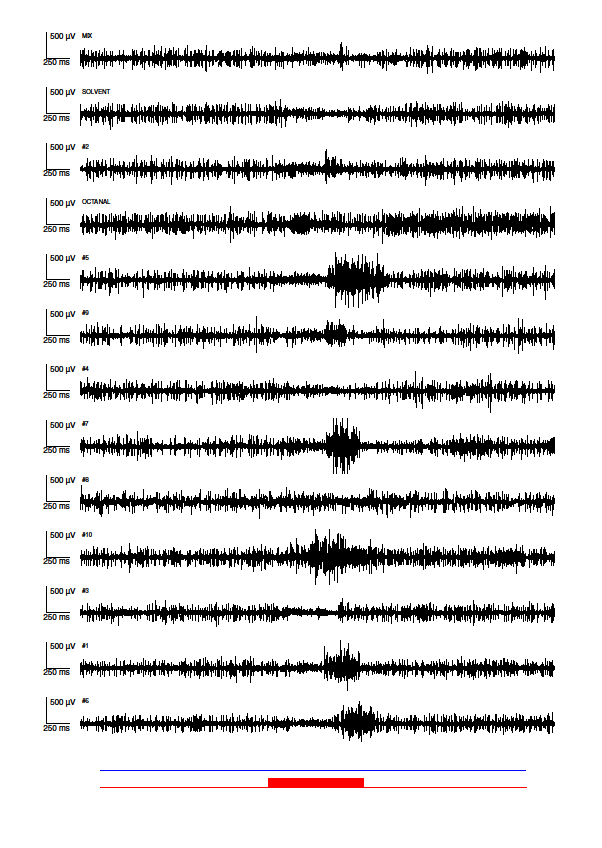
**

**
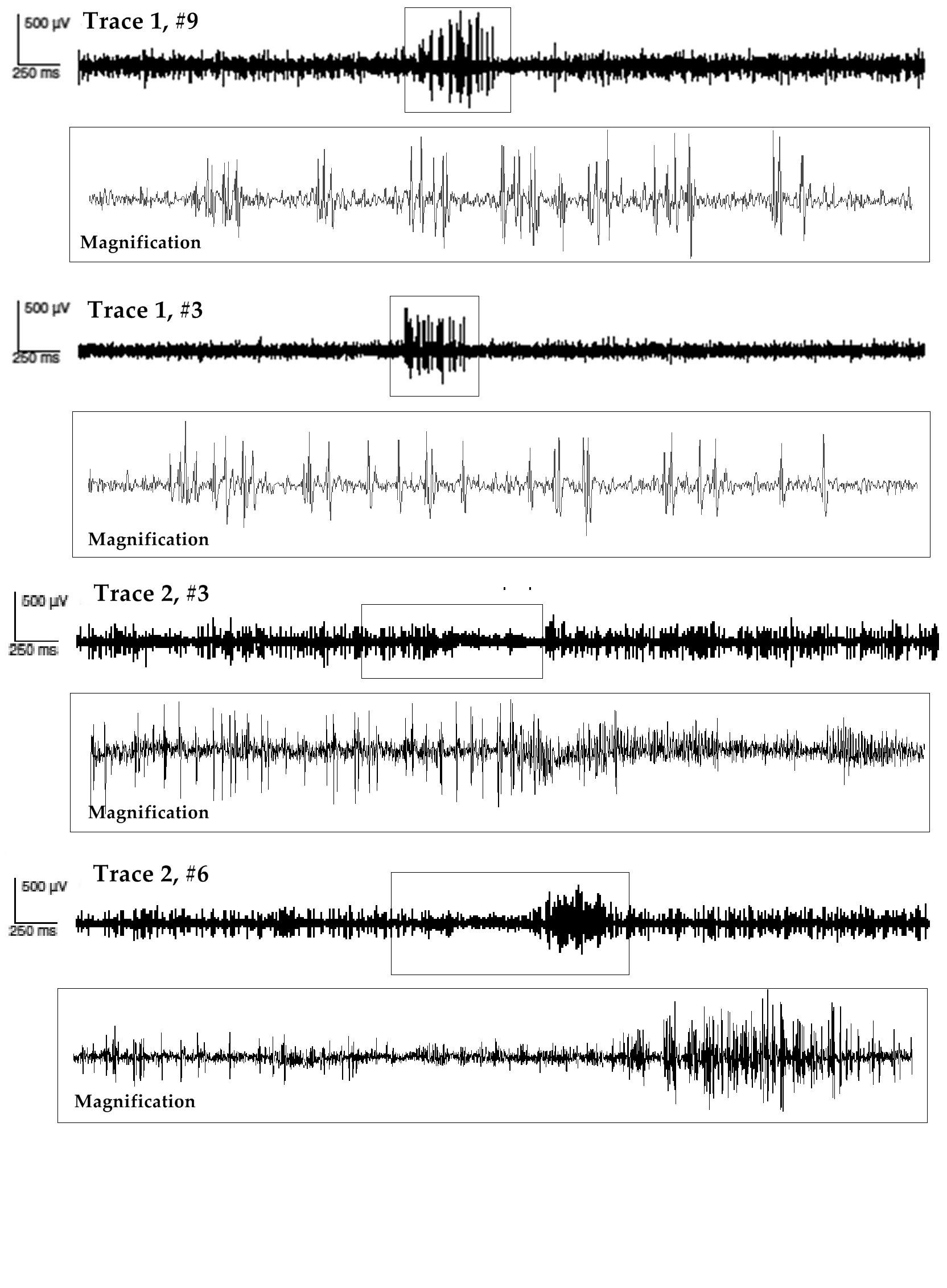
**
